# The Oncogenic Role of Prostate Stem Cell Antigen (PSCA) in Colorectal Cancer: Implications for Targeted Therapy

**DOI:** 10.3390/cimb48070737

**Published:** 2026-07-20

**Authors:** Jinyue Duan, Yi Wang, Qisen Li, Yujue Wang, Jinrui Liu, Yi Qi, Yichi Zhang, Changhao Fu, Zhongyi Cong, Can Wang, Manman Su

**Affiliations:** 1 Department of Regenerative Medicine, School of Pharmaceutical Sciences, Jilin University, Changchun 130012, China; 2 Department of Physiology, School of Basic Medical Sciences, Beihua University, Jilin 132013, China; 3 VA Palo Alto Health Care System, Medical School, Stanford University, Palo Alto, CA 94304, USA

**Keywords:** cancer stem cells, colorectal cancer, MAPK, PSCA, siRNA

## Abstract

Prostate stem cell antigen (PSCA), a pivotal member of the lymphocyte antigen-6 (Ly6) protein family, has been implicated in the tumorigenesis and neoplastic progression of diverse cancer types. In this study, we conducted a thorough investigation into the role of PSCA in the development of colorectal cancer (CRC). Survival analysis based on The Cancer Genome Atlas (TCGA) dataset demonstrated that elevated expression of PSCA was tightly correlated with unfavorable overall survival, inferior relapse-free survival, and worse post-progression survival among CRC patients. Additionally, PSCA exhibited significantly higher expression levels in colorectal cancer stem cell (CRC-SCs) relative to CRC cell lines. Loss-of-function assays using small interfering RNA (siRNA)-mediated silencing were performed to evaluate the effects of PSCA downregulation on the stemness properties of CRC-SCs, including proliferative capacity, invasive potential, and apoptotic rate, which were assessed by MTS assay, transwell invasion assay, and flow cytometry analysis, respectively. The results showed that silencing PSCA markedly suppressed the proliferation and invasion of CRC-SCs, while significantly promoting cellular apoptosis. RNA sequencing was performed to identify differentially expressed genes (DEGs) in the PSCA knockdown group compared to the negative control group. Follow-up analyses using Gene Ontology (GO) and Kyoto Encyclopedia of Genes and Genomes (KEGG) indicated that these DEGs were significantly enriched in the cell-substrate adherens junction term and the mitogen-activated protein kinase 9MAPK signaling pathway. Moreover, PSCA silencing substantially reduced the phosphorylation levels of the core MAPK signaling constituents, pBRAF and pERK1/2; conversely, PSCA overexpression prominently upregulated the expression of pBRAF and pERK1/2. In nude mice with CRC-SCs cancer xenograft tumors, treatment with PSCA siRNA significantly decreased tumor volume and weight, while also notably extending the survival time of the tumor-bearing mice compared to the control group. Collectively, these findings confirm that PSCA plays a critical oncogenic role in CRC cancer growth and malignant progression, suggesting its potential as a novel and promising therapeutic target for CRC.

## 1. Introduction

Colorectal cancer (CRC) has long been considered a widespread malignant neoplasm, and its incidence has increased among young people in recent years [1,2]. Most patients with advanced CRC suffer from liver and/or lung metastases, resulting in an unfavorable prognosis [3,4,5,6]. As scientific research advances in depth, a growing number of therapeutic targets for CRC have been identified, including various proteins, long noncoding RNAs (lncRNAs), and microRNAs (miRNAs); however, there is still no effective treatment for CRC at present [7,8,9]. Consequently, it is essential to identify new therapeutic targets and elucidate the molecular mechanisms involved in CRC progression.

Malignancy is intricately associated with tumor heterogeneity. Cancer stem cells (CSCs), a minor subset of cancer cells linked to this heterogeneity, possess the abilities of self-renewal, differentiation, and tumor initiation. These properties allow CSCs to mediate drug resistance and cancer progression, thereby triggering tumor recurrence and distant metastasis [10,11,12,13,14]. Furthermore, CSCs have high plasticity, enabling them to differentiate into diverse cell populations. They also demonstrate strong resistance to harsh conditions in the tumor microenvironment and chemotherapeutic agents and can invade healthy tissues [14,15,16,17,18,19]. Targeting CSCs is a promising strategy to enhance the prognosis of patients with CRC [20,21]. Colorectal cancer stem cells (CRC-SCs) are the initiating cells of CRC and are deemed to promote CRC occurrence, proliferation, and metastasis [22,23,24]. Thus, exploring the mechanisms through which CRC-SCs initiate CRC is crucial for identifying targets for the curative treatment of CRC.

The lymphocyte antigen-6 (Ly6) superfamily is a large protein group that comprises a subfamily of glycosylphosphatidylinositol (GPI)-anchored membrane proteins, including Ly6a, Ly6d, Ly6e, PSCA, and Lynx1. In recent years, these proteins have drawn increasing attention due to their potential involvement in tumor progression [25,26]. Aberrantly expressed in pituitary and lung malignancies, Ly6a is particularly recognized as a biomarker of lung tumor initiation [27,28]. However, Ly6d plays dichotomous roles, acting as a tumor suppressor in prostate cancer yet an oncogenic driver in squamous cell carcinoma [29,30,31]. Furthermore, Ly6e accelerates CRC progression [32,33], while Lynx1 promotes the development of pancreatic ductal cancer [34].

Prostate stem cell antigen (PSCA) is an oncogene that resides on chromosome 8q24.3, a chromosome harboring a variety of other oncogenes [35,36,37]. In prostate and ovarian cancers, PSCA is aberrantly overexpressed and exhibits a strong positive correlation with poor patient prognosis [38,39,40]. Moreover, increased PSCA expression is associated with more advanced cancer stages and higher Gleason scores in prostate cancer [38,41]. However, the correlation between PSCA and CRC has not been fully elucidated. Growing evidence indicates that PSCA can drive oncogenic phenotypes by upregulating c-Myc across multiple epithelial malignancies [42]. The transcription factor c-Myc acts as a core upstream regulator of the core pluripotency transcription factors Oct-4, Sox-2, and Nanog; together, this quartet forms a coordinated transcriptional network that maintains self-renewal, sphere-forming capacity, and stemness signatures in colorectal cancer stem cells [43]. Aberrantly elevated c-Myc directly activates the promoters of Oct-4, Sox-2, and Nanog in CRC cell lines, thereby sustaining the malignant stem-like state and promoting metastasis and chemoresistance [44]. Although the individual oncogenic functions of PSCA, c-Myc, and these pluripotency markers have been separately documented in the previous literature, whether PSCA remodels colorectal cancer stem cell properties through the c-Myc/Oct-4/Sox-2/Nanog regulatory axis remains poorly characterized. Accordingly, we detected the above pluripotency markers across our constructed cell models to dissect the potential PSCA-mediated molecular cascade governing CRC stemness [45]. This study was designed to explore the relationship between PSCA and CRC.

To identify an appropriate in vitro model for investigating PSCA-mediated stemness and malignant progression, we screened multiple classic human colorectal cancer cell lines and finally selected HCT116 and SW620 for subsequent research. Western blot screening revealed that HCT116 exhibits the highest endogenous PSCA expression level among all detected cell lines including HT29, COLO205, SW480, and SW620, while SW620 presents relatively low basal PSCA abundance. The distinct endogenous PSCA expression levels between the two cell lines provide ideal contrasting conditions to explore the biological function of PSCA, making them suitable models to dissect how PSCA regulates CRC stem-like phenotypes and its downstream signaling cascades.

Multiple complementary experimental systems were adopted to systematically dissect the biological function and molecular mechanism of PSCA in colorectal cancer stem cells. Gain- and loss-of-function cell models constructed using PSCA overexpression plasmids and siRNA interference were applied to establish the causal link between PSCA and malignant stem-like phenotypes. A panel of functional assays, including sphere formation, MTS, transwell, and apoptosis assays, were conducted to evaluate self-renewal, proliferation, invasion, and chemoresistance. RNA-seq, coupled with GO and KEGG enrichment analyses, was used to preliminarily screen differentially altered signaling cascades, and Western blot further validated the expression changes of core regulatory proteins. Finally, subcutaneous xenograft models in nude mice were established to verify the tumor-suppressive effect of siPSCA at the in vivo level. This integrated in vitro and in vivo research strategy represents a standard and rigorous experimental framework for investigating gene regulatory networks in CRC stem cell studies, allowing comprehensive and robust characterization of PSCA-mediated oncogenic signaling.

## 2. Materials and Methods

### 2.1. Cells Culture

Human colorectal cancer cell lines (COLO 205, HT-29, HCT116, SW480, and SW620) were obtained from the Cell Bank of the Chinese Academy of Sciences (CAS, Shanghai, China) and cultured in RPMI 1640 medium (Gibco, Thermo Fisher Scientific, Bohemia, NY, USA) supplemented with 10% fetal bovine serum (FBS). Cells were cultured in the incubator at 37 °C and 5% CO_2_ air atmosphere.

CD44^+^CD326^+^ colorectal cancer stem cells (HCT116SCs derived from HCT116 and SW620SCs derived from SW620) were cultured in serum-free DMEM/F12 medium (Gibco, Thermo Fisher Scientific, Bohemia, NY, USA). The culture medium was supplemented with 20 ng/mL epidermal growth factor (EGF, Invitrogen, Thermo Fisher Scientific, VNO, Vilnius, Lithuania), 20 ng/mL basic fibroblast growth factor (bFGF, Invitrogen, Carlsbad, CA, USA), and 1× B27 serum-free supplement (Gibco, Thermo Fisher Scientific, Bohemia, NY, USA). Cells were maintained in a humidified atmosphere at 37 °C and 5% CO_2_ to form tumor spheres. The medium was refreshed with growth factors once every three days to sustain stem cell properties.

### 2.2. Magnetic-Activated Cell Sorting (MACS)

CD44^+^CD326^+^ colorectal cancer stem cells were isolated via immunomagnetic bead sorting following the standard protocol of the CELLection Biotin Binder kit (Invitrogen, Thermo Fisher Scientific, VNO, Vilnius, Lithuania). In short, digested single-CRC-cell suspensions (4.0 × 10^7^ cells) were collected and resuspended in separation buffer matched with the kit. Cells were mixed with Dynabeads^®^ conjugated to anti-CD44 antibody (Invitrogen, Thermo Fisher Scientific, VNO, Vilnius, Lithuania) and incubated at 4 °C with slow shaking for 20 min. After incubation, test tubes were placed on magnetic stands to retain CD44^+^ cells combined with magnetic beads, while unmarked negative cells were removed by absorbing the supernatant. Captured CD44^+^ cells were dissociated from Dynabeads according to kit instructions to obtain a pure suspension of CD44^+^ CRC cells.

For secondary sorting, the enriched CD44^+^ cell population was incubated with anti-CD326 antibody-coated Dynabeads^®^ under identical incubation conditions (4 °C, rotating for 20 min). The same magnetic separation and bead-release procedures described above were repeated to isolate the CD326^+^ subpopulation from CD44^+^ cells, yielding purified double-positive CD44^+^CD326^+^ CRC cells for subsequent sphere culture.

### 2.3. Cell Transfection

PSCA and negative control (NC) siRNAs were obtained from Ribo Bio (Guangzhou, China). The PSCA siRNA sequences were as follows: siPSCA#1 (5′-TCRCTGACRCGTCATCAGCAA-3′), PSCA siRNA#2 (5′-GCTTGAACTGCGTGGATGA-3′), and PSCA siRNA#3 (5′-GTGGGCAAGAAGAACATCA-3′). pc-DNA3.1 empty vector and pc-DNA3.1-PSCA (pc-PSCA) overexpression plasmid were provided by the Public Protein/Plasmid Library (PPL; Nanjing, China). All plasmids were purified with endotoxin-free extraction kits prior to transfection to avoid cytotoxicity.

Three-day cultured CR-CSC tumor spheres were digested into single-cell suspensions. Cells were counted and seeded in six-well plates at a density of (1.0 × 10^6^) cells/well with antibiotic-free CR-CSC medium. Transfection was conducted using Hieff Trans™ Liposomal Transfection Reagent (Yeasen, Shanghai, China) in accordance with the manufacturer’s protocol. To elaborate, siRNA fragments or plasmid DNA, together with liposome transfection reagent, were diluted separately with Opti-MEM medium. After uniform mixing and 5 min standing at room temperature to form transfection complexes, the mixture was slowly added to cell layers. Cells were incubated at 37 °C under 5% CO_2_ and harvested 48 h later for subsequent experiments. The experimental groups were set as follows: Blank group, cells without siRNA and transfection reagent; Mock group, cells treated with transfection reagent only without siRNA or plasmid; NC group, cells transfected with transfection reagent plus negative control siRNA (for knockdown) or empty pc-DNA3.1 plasmid and pc-DNA3.1-PSCA plasmid (for overexpression).

### 2.4. Cell Proliferation

#### 2.4.1. MTS Assay

Cell proliferation was quantified using a commercial MTS assay kit purchased from Promega Corporation (Madison, WI, USA). In brief, cells treated with siRNA or plasmids following the protocol in Section 2.3 were resuspended and evenly planted into 96-well plates at 1.0 × 10^4^ cells per well using antibiotic-free CRC-SC medium. Six duplicate wells were set for each group to minimize experimental deviation. After 48 h of routine incubation at 37 °C and 5% CO_2_, 20 μL MTS working solution was added to each well. The plates were incubated for an additional 2 h in the incubator protected from light. The absorbance value (OD value) of each well was recorded at a wavelength of 450 nm using a microplate reader to reflect relative cell viability and proliferation capacity.

#### 2.4.2. Colony Formation Assay

Transfected single-cell suspensions were counted, and 1.0 × 10^3^ viable cells were inoculated into each 6 cm culture dish with 5 mL RPMI 1640 medium (Gibco, Thermo Fisher, Grand Island, NY, USA) supplemented with 10% fetal bovine serum (FBS, Gibco, Thermo Fisher). Cells were maintained in a humidified incubator at 37 °C with 5% CO_2_. The full culture medium was replaced with fresh medium every 3 days to sustain cell growth. After continuous culture for 14 days, visible cell colonies were formed. The culture medium was discarded, and cells were washed gently with pre-cooled phosphate-buffered saline (PBS). Cells were fixed with 4% paraformaldehyde (Biosharp, Hefei, China) at room temperature for 15 min, followed by rinsing with PBS again. Fixed colonies were stained with 1% crystal violet solution (Solarbio Life Sciences, Beijing, China) for 10 min. Excess staining solution was washed off with running water, and plates were air-dried naturally for subsequent colony counting and imaging analysis.

### 2.5. Pharmacological Rescue of ERK1/2 Activity

Tert-butyl hydroquinone (TBHQ, MedChemExpress, Monmouth Junction, NJ, USA) was utilized as a specific activator of ERK1/2 phosphorylation in this study. At 6 h after cell transfection, a final concentration of 60 µM TBHQ was added to the cell culture medium. The cells were continuously cultured for another 42 h under standard incubator conditions (37 °C, 5% CO_2_) to complete ERK1/2 pathway activation and then harvested for subsequent functional and molecular detection experiments.

### 2.6. Cell Apoptosis

Cell apoptotic rates were quantitatively detected at 48 h post-transfection using the FITC-Annexin V/PI apoptosis detection kit (Becton Dickinson and Company, San Diego, CA, USA). Simply put, roughly 1.0 × 10^5^ cells were collected from every group. Cell precipitates were washed twice with pre-cooled 1× PBS to eliminate residual culture medium and cell fragments. Cells were resuspended in binding buffer and double-stained with 5 μL FITC-Annexin V and 5 μL PI working fluid. After incubation for 15 min at room temperature in the dark, cell apoptosis was analyzed by flow cytometry within 1 h to ensure the accuracy of fluorescence signals. All apoptosis experiments were repeated at least three times independently.

### 2.7. Cell Invasion

Cell invasive ability was evaluated using 8 μm pore-size Transwell invasion chambers (Corning, Corning, NY, USA) pre-coated with uniform Matrigel matrix (Becton Dickinson and Company, San Diego, CA, USA). Prior to seeding, transfected cells were starved in serum-free DMEM/F12 medium. Then, 1.0 × 10^5^ cells suspended in 100 μL serum-free DMEM/F12 medium were added to the upper compartment of chambers. Meanwhile, 500 μL DMEM/F12 complete medium containing 10% FBS was poured into the lower chamber to serve as chemotactic inducer. After 48 h of routine culture, the non-invasive cells remaining on the upper membrane surface were gently wiped off. The invasive cells on the lower membrane were fixed and stained with crystal violet solution (Solarbio Life Sciences, Beijing, China). For quantitative analysis, three random and independent visual fields per chamber were photographed under a microscope, and the number of invasive cells was counted for statistical analysis. All invasion assays were performed in triplicate.

### 2.8. RNA-Seq and Bioinformatics Analysis

#### 2.8.1. RNA-Seq

Cells transfected with negative control siRNA and PSCA siRNA were collected separately, and TRIzol reagent (Invitrogen) was adopted to extract total RNA following official product instructions. RNA integrity and purity were detected before constructing sequencing libraries. Sequencing libraries were built using the NEBNext^®^ Ultra™ RNA Library Prep Kit for Illumina^®^ (NEB; Ipswich, MA, USA), and unique index barcodes were added to each sample for read classification. Cluster amplification was performed on a cBot Cluster Generation System with the TruSeq PE Cluster Kit v3-cBot-HS (Illumina, San Diego, CA, USA), followed by paired-end RNA sequencing on the Illumina platform.

All raw RNA-seq sequencing data generated in this study have been deposited into the NCBI Gene Expression Omnibus (GEO) database under accession number GSE337374, which is publicly accessible for further validation.

#### 2.8.2. Gene Ontology (GO) and Kyoto Encyclopedia of Genes and Genomes (KEGG) Enrichment Analyses

Differentially expressed genes (DEGs) between the NC and siPSCA groups were subjected to GO enrichment analysis with the clusterProfiler R package. GO entries are divided into three categories: biological process (BP), cellular component (CC), and molecular function. GO terms with *p* < 0.05 and log_2_∣FC∣ > 0.5 were regarded as significantly enriched.

The Kyoto Encyclopedia of Genes and Genomes (KEGG, http://www.genome.jp/kegg/ (accessed on 23 September 2022)) database was used to perform enrichment analysis of DEGs between the two groups via the clusterProfiler R package (R version 4.2.1). KEGG pathways with *p* < 0.05 and log_2_∣FC∣ > 0.5 were defined as significantly enriched pathways.

### 2.9. Western Blotting

Total cellular protein of each experimental group was extracted with RIPA lysis buffer (DINGGUO, Beijing, China) pre-mixed with fresh protease inhibitor cocktail (Beyotime Biotechnology, Shanghai, China). Protein samples with consistent loading quantity were separated via SDS-PAGE gel electrophoresis and electrically transferred onto PVDF membranes. Membranes were sealed with 5% skim milk without fat at room temperature for one hour to block non-specific binding sites. The membranes were then incubated overnight at 4 °C with primary antibodies against target proteins, including anti-PSCA (Abcam), anti-BRAF, anti-pBRAF, anti-ERK1/2, anti-pERK1/2 (Cell Signaling Technology), and anti-GAPDH (Beyotime Biotechnology) as a loading control, all at a dilution of 1:1000. After extensive washing with TBST, the membranes were incubated with horseradish peroxidase (HRP)-conjugated anti-rabbit IgG secondary antibody (1:2000, Beyotime Biotechnology) for 1 h at 25 °C. Protein bands were visualized using an enhanced chemiluminescence (ECL) detection reagent (Yeasen, Shanghai, China) and imaged with a Tanon Imaging System (Tanon, Shanghai, China). All Western blot experiments were independently repeated at least three times (biological replicates).

### 2.10. Animals

Six-week-old SPF-grade male BALB/c nude mice without thymus were bought from Beijing HFK Bioscience Co., Ltd., Beijing, China. Animals were raised in standardized SPF animal rooms, where temperature stayed steady at 22–25 °C with a 12 h light–dark shift. Sterile water and conventional mouse feed were supplied freely during the whole experiment. All animal operations and in vivo experimental schemes followed the unified animal welfare standards authorized by the Animal Care Committee of Jilin University (Ethics Certificate No. 20210065).

For the in vivo xenograft experiment, mice were randomly divided into three experimental groups, including a Blank group, NC group, and siPSCA group, with 5 mice per group to ensure biological reproducibility.

### 2.11. Xenograft Assay

All animal experiments were carried out in compliance with the protocols approved by the institutional animal care and use committee. For the in vivo subcutaneous xenograft tumor model, 5.0 × 10^5^ HCT116 stem cells (HCT116SCs) were resuspended in sterile phosphate-buffered saline (PBS) and subcutaneously injected into the right flank of each BALB/c nude mouse. When the subcutaneous tumor volume reached approximately 50 mm^3^, mice with uniform tumor size were randomly assigned into two groups. Intratumoral injection of NC siRNA or PSCA siRNA was performed three times per week for a continuous three weeks.

The body weight of each mouse and tumor dimensions were measured every other day at a fixed time point. Tumor volume was calculated using the formula V = (a × b^2^)/2, where a represents the maximum tumor length and b represents the maximum tumor width measured by a digital caliper.

### 2.12. Histological Staining and Histomorphological Analysis

Tumor tissues separated from xenograft mice were fixed in 4% paraformaldehyde immediately, followed by standard dehydration and paraffin embedding to make tissue wax blocks. Continuous paraffin slices with a thickness of 4 μm were prepared for hematoxylin-eosin (HE) and immunohistochemistry (IHC) staining.

For HE staining: Tissue sections were deparaffinized in xylene and rehydrated through a graded ethanol series. After hematoxylin nuclear staining and eosin cytoplasmic counterstaining, sections were dehydrated, cleared, and mounted for microscopic observation.

For IHC staining: Deparaffinized and rehydrated sections were treated with 3% H_2_O_2_ for 30 min at room temperature to block endogenous peroxidase activity. Antigen retrieval was performed by microwave heating in 10 mM citrate buffer (pH 6.0) for 15 min, and then sections were cooled naturally to room temperature. After blocking non-specific binding sites, slides were incubated overnight at 4 °C with primary antibodies diluted at 1:100, including PSCA (Abcam), pERK1/2, Ki67, Bcl-2, Bax, caspase-3, caspase-7, E-cadherin, N-cadherin, vimentin, and ZEB1 (CST). The next day, sections were incubated with biotin-conjugated secondary antibody and streptavidin working solution sequentially. DAB Horseradish Peroxidase Color Development Kit (Beyotime Biotechnology) was applied for chromogenic reaction under visual monitoring. All stained slices were counterstained with hematoxylin, dehydrated, and sealed for image acquisition and quantitative analysis.

### 2.13. Statistical Analysis

GraphPad Prism 9.5.1 (GraphPad Software, San Diego, CA, USA) was used for all statistical evaluations. All quantitative data were expressed as mean ± SEM. The Shapiro–Wilk test was utilized to assess the normal distribution of each dataset prior to parametric statistical comparison. Only data with normal distribution were analyzed by two-tailed Student’s *t*-test and two-way analysis of variance (ANOVA), which supports the rational application of parametric tests in this work. Quantitative information, including biological replicates of cellular experiments and the exact number of mice in each xenograft group, has been clearly described in the corresponding method subsections above. Differences with *p* < 0.05 were considered statistically significant.

## 3. Results

### 3.1. PSCA Expression in CRC and Isolation, Identification of CRC-SCs

**Figure 1:**
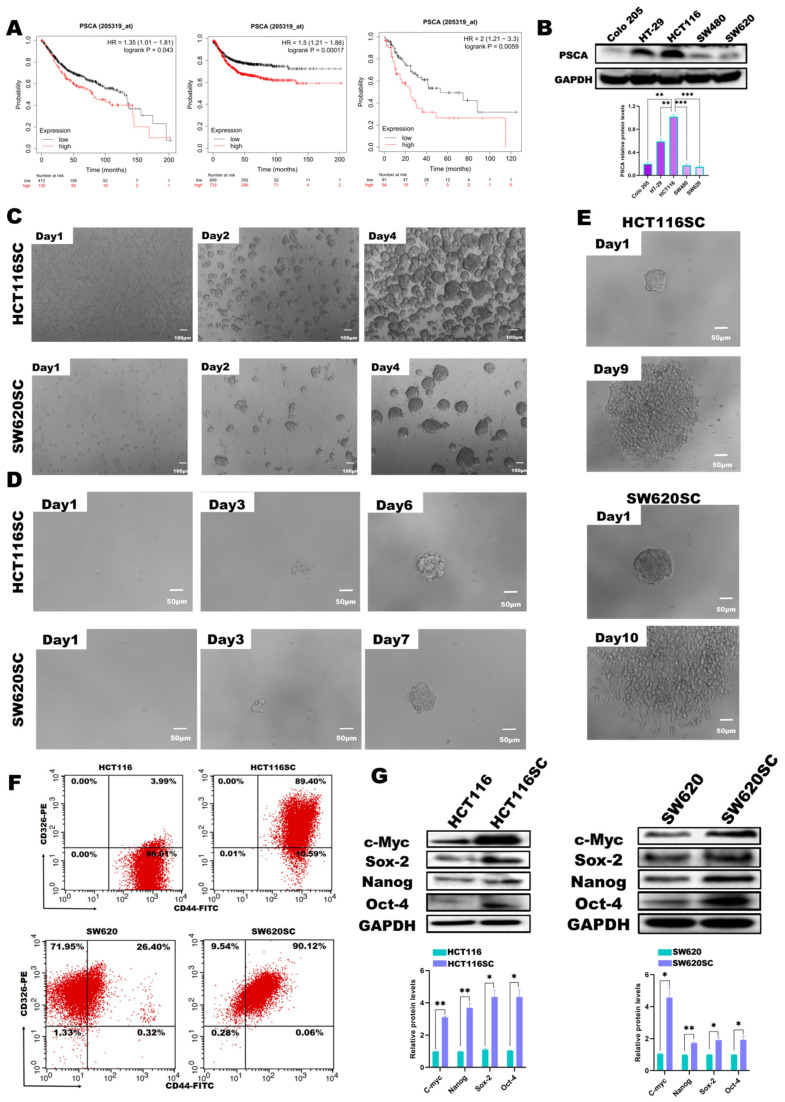
PSCA expression in CRC and isolation, identification of CRC-SCs. (**A**) Survival curves generated by Kaplan–Meier plotter illustrating the overall survival, relapse-free survival, and post-progression survival of CRC patients stratified by high and low PSCA expression levels. (**B**) Relative PSCA protein expression in CRC cell lines assessed by Western blotting (** *p* < 0.01, *** *p* < 0.001). (**C**) In vitro culture of HCT116SCs and SW620SCs in serum-free DMEM/F12 medium for 4 days. (**D**) Tumor sphere formation from HCT116SCs and SW620SCs. (**E**) Differentiation of CRC-SCs into adherent cells induced by medium supplemented with 10% FBS. (**F**) Flow cytometric analysis of CD44 and CD326 expression in CRC-SCs and parental CRC cells. (**G**) Western blotting analysis of c-Myc, Sox-2, Nanog, and Oct-4 expression in CRC-SCs and parental CRC cells (* *p* < 0.05, ** *p* < 0.01).

We utilized the online Kaplan–Meier Plotter survival database (https://kmplot.com/analysis/ (accessed on 21 May 2023)) to systematically investigate the clinical correlation between PSCA expression and long-term survival outcomes among human colorectal cancer patients. Our bioinformatic analysis clearly demonstrated that aberrantly elevated PSCA expression is tightly associated with unfavorable clinical prognosis in CRC cohorts. Specifically, patient subgroups with high PSCA expression displayed remarkably worse overall survival, relapse-free survival, and post-progression survival curves when compared to patients carrying low PSCA levels (Figure 1A). Subsequently, we performed Western blot detection to quantify endogenous PSCA protein abundance across a panel of common CRC cell lines. The detection results illustrated that PSCA expression reached the highest level in HCT116 cells, while SW620 cells presented the lowest endogenous PSCA expression (Figure 1B).

To further characterize the endogenous expression profile of PSCA in colorectal cancer stem cells (CRC-SCs), we isolated CRC-SC subpopulations from wild-type HCT116 and SW620 cells by magnetic-activated cell sorting (MACS) using CD44 and CD326 as surface markers [46,47,48], designated as HCT116SCs and SW620SCs, respectively. Flow cytometry was performed to quantitatively assess the proportion of CD44^+^CD326^+^ cells in the sorted populations. The results confirmed that the proportion of CD44^+^CD326^+^ cells was significantly higher in the sorted CRC-SC groups than in the unsorted parental CRC cells, indicating the successful isolation of CRC-SC subpopulations via MACS (Figure 1F).

Following four consecutive days of serum-free in vitro incubation, the purified CRC-SCs spontaneously formed round, compact tumor spheres under suspension culture conditions (Figure 1C). Notably, even a single isolated HCT116SC or SW620SC cell possessed sufficient self-renewal capacity to independently develop an intact, viable tumor sphere (Figure 1D). Moreover, the primary tumor spheres generated from HCT116SCs and SW620SCs retained intact multi-lineage differentiation potential and were able to convert into adherent epithelial tumor cells under appropriate adherent induction culture (Figure 1E). In parallel, quantitative Western blotting experiments further verified that the protein expression of canonical stemness markers (c-Myc, Nanog, Sox-2, and Oct-4) was significantly upregulated in both types of CRC-SCs when matched with their corresponding parental cell lines (Figure 1G).

### 3.2. PSCA Is Highly Expressed in CRC-SCs and Modulates Stemness-Associated Transcription Factors

**Figure 2:**
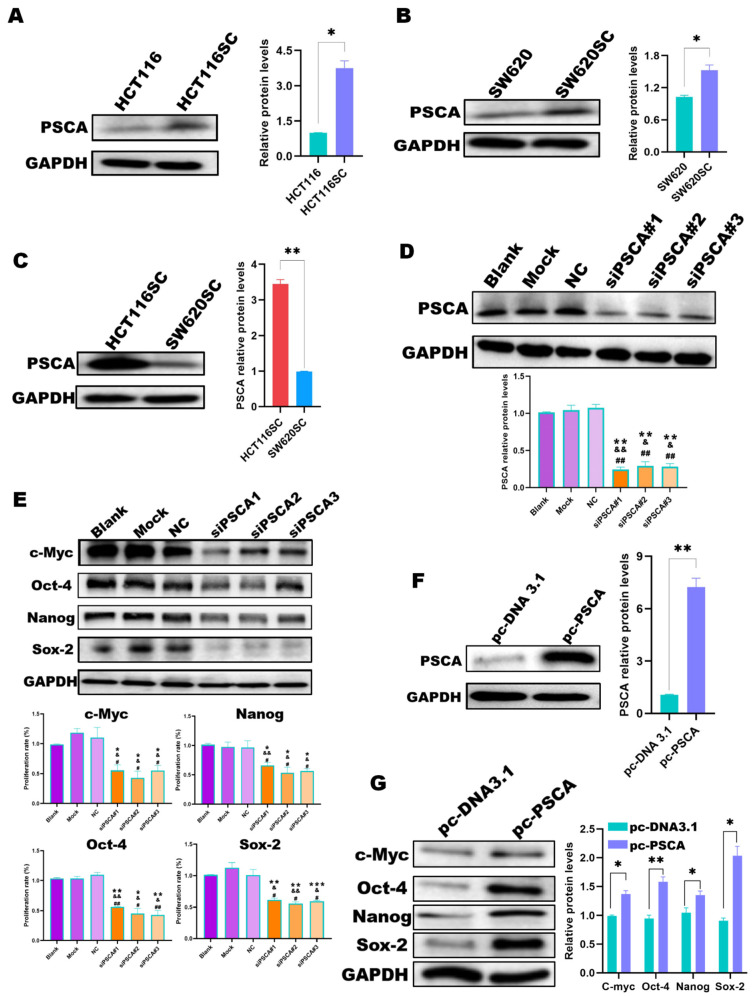
High PSCA expression in CRC-SCs regulates stemness-associated transcription factors. (**A**) PSCA protein expression in HCT116 cells and HCT116SCs (* *p* < 0.05). (**B**) PSCA protein levels in SW620 cells and SW620SCs (* *p* < 0.05 vs. SW620). (**C**) PSCA protein expression in HCT116SCs and SW620SCs (** *p* < 0.01). (**D**) PSCA protein levels in HCT116SCs after siPSCA-mediated knockdown (** *p* < 0.01 vs. Blank; & *p* < 0.05, && *p* < 0.01 vs. Mock; ## *p* < 0.01 vs. NC). (**E**) Expression of stemness transcription factors (c-Myc, Oct-4, Nanog, and Sox-2) after PSCA knockdown (* *p* < 0.05, ** *p* < 0.01, *** *p* < 0.001 vs. Blank; & *p* < 0.05, && *p* < 0.01 vs. Mock; # *p* < 0.05, ## *p* < 0.01 vs. NC). (**F**) PSCA protein levels in SW620SCs after pcDNA3.1-PSCA-mediated overexpression (** *p* < 0.01 vs. pcDNA3.1 control). (**G**) Expression of c-Myc, Oct-4, Nanog, and Sox-2 after PSCA overexpression.(* *p* < 0.05, ** *p* < 0.01).

To clarify the differential expression pattern of PSCA between colorectal cancer bulk cells and their corresponding stem cell subpopulations, we performed Western blotting to quantify endogenous PSCA protein levels in paired HCT116 parental cells and HCT116SCs. Quantitative gray-scale analysis of blotting bands revealed that the protein abundance of PSCA was remarkably and statistically higher in HCT116SCs than in conventional adherent HCT116 cells (Figure 2A). Parallel Western blotting detection was also conducted using SW620 parental cells and sorted SW620SCs, which yielded consistent results: SW620SCs exhibited markedly elevated PSCA expression compared with unsorted SW620 cells (Figure 2B).

We next made a direct comparison of PSCA expression between the two CRC stem cell lines. The quantitative immunoblot data showed that HCT116SCs possessed significantly stronger PSCA protein signals than SW620SCs (Figure 2C). Collectively, these expression differences implied that PSCA may exert critical regulatory functions in maintaining the malignant stem-like characteristics of CRC-SCs. Accordingly, we constructed two sets of cell models to explore the biological function of PSCA: PSCA knockdown models in HCT116SCs and PSCA overexpression models in SW620SCs.

Three independent siRNA sequences targeting PSCA (siPSCA1, siPSCA2, siPSCA3) were separately transfected into HCT116SCs, with Blank, Mock-transfected, and negative control (NC) groups set as parallel controls. Western blotting was performed to detect PSCA and stemness transcription factors (c-Myc, Oct-4, Nanog, and Sox-2) protein levels. The band quantification results illustrated that all three siPSCA fragments effectively suppressed endogenous PSCA expression; meanwhile, the expression of the four stemness-related transcriptional regulators was synchronously and significantly decreased relative to all three control groups (Figure 2D,E).

For gain-of-function verification, the overexpression plasmid pc-DNA3.1-PSCA (abbreviated as pc-PSCA) and empty vector pc-DNA3.1 were separately delivered into SW620SCs. Immunoblotting results validated that ectopic PSCA overexpression successfully boosted PSCA protein abundance. Correspondingly, the expression of c-Myc, Oct-4, Nanog, and Sox-2 was simultaneously upregulated in the pc-PSCA group versus the empty vector control group (Figure 2F,G). Taken together, these loss- and gain-of-function assays demonstrated that PSCA positively regulates the expression of key stemness transcription factors in CRC-SCs.

### 3.3. PSCA Modulates the Stemness of CRC-SCs, Encompassing Their Proliferation, Invasion, and Apoptosis

As demonstrated in the above immunoblotting results, silencing endogenous PSCA in HCT116SCs markedly suppressed the expression levels of core stemness transcription factors. To further elaborate the functional influence of PSCA depletion on multiple malignant stem-like phenotypes of HCT116SCs, we systematically examined three key biological characteristics closely associated with tumor stemness, namely cell proliferative activity, apoptotic level, and cellular invasive potential.

**Figure 3:**
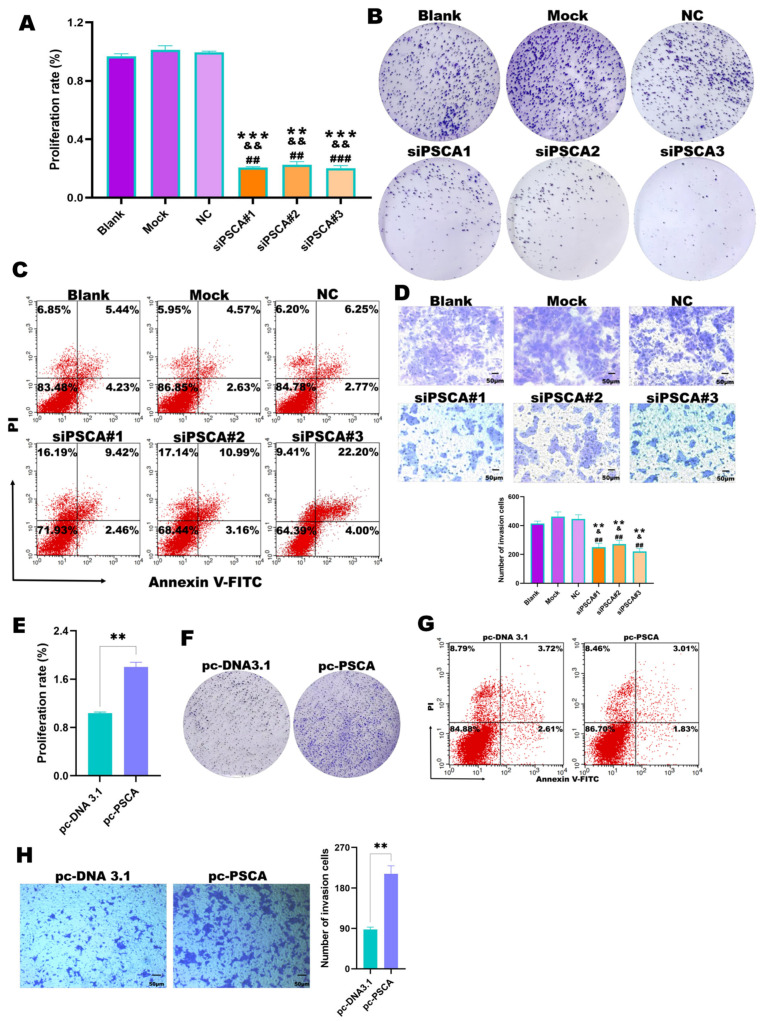
In vitro biological behavior assays of PSCA in CRC-SCs. (**A**,**B**) MTS and colony formation assays showing proliferation of HCT116SCs after siPSCA-mediated knockdown (** *p* < 0.01, *** *p* < 0.001 vs. Blank; && *p* < 0.01 vs. Mock; ## *p* < 0.01, ### *p* < 0.001 vs. NC). (**C**) Flow cytometric analysis of apoptotic HCT116SCs after siPSCA transfection using Annexin V-FITC/PI staining. (**D**) Representative images and quantification of invaded HCT116SCs in the Transwell assay after siPSCA transfection (** *p* < 0.01 vs. Blank; & *p* < 0.05 vs. Mock; ## *p* < 0.01 vs. NC). (**E**,**F**) MTS and colony formation assays showing proliferation of SW620SCs after pcDNA3.1-PSCA-mediated overexpression (** *p* < 0.01 vs. pcDNA3.1). (**G**) Flow cytometric analysis of apoptotic SW620SCs after pcDNA3.1-PSCA transfection using Annexin V-FITC/PI staining. (**H**) Representative images and quantification of invaded SW620SCs in the Transwell assay after pcDNA3.1-PSCA transfection (** *p* < 0.01 vs. pcDNA3.1).

Quantitative detection via MTS colorimetric viability assay and plate colony formation assay consistently displayed that the proliferative activity of HCT116SCs was remarkably impaired upon PSCA knockdown relative to the Blank, Mock, and NC control groups (Figure 3A,B). Flow-cytometry-based Annexin V/PI double staining was subsequently applied to quantitatively measure cellular apoptosis. Statistical analysis of flow cytometric data uncovered a prominent elevation in the overall apoptotic level of HCT116SCs after PSCA silencing compared with all control counterparts (Figure 3C). Matrigel-precoated Transwell invasion chambers were next utilized to evaluate cell invasive ability. Manual counting of stained penetrating cells under a microscope verified that PSCA knockdown substantially weakened the invasive capacity of HCT116SCs (Figure 3D).

To reciprocally validate the positive regulatory role of PSCA on CRC-SC stemness, we constructed a PSCA overexpression cell model in SW620SCs and detected changes in cell proliferation, apoptosis, and invasive capacity. MTS and colony formation assays illustrated that forced exogenous overexpression of PSCA significantly accelerated the proliferative rate of SW620SCs when compared with cells transfected with blank pc-DNA3.1 empty vector control (Figure 3E,F). In contrast, flow cytometric apoptosis detection suggested no obvious statistical discrepancy in overall apoptotic levels between the PSCA-overexpressing group and empty vector control group (Figure 3G). This phenomenon may be attributed to the inherently low basal apoptotic level of wild-type SW620SCs, leaving limited room for further anti-apoptotic improvement upon exogenous PSCA overexpression. Meanwhile, Transwell invasion tests confirmed that ectopic PSCA expression dramatically strengthened the invasive capability of SW620SCs versus empty vector control cells (Figure 3H).

### 3.4. PSCA Modulates the Stemness of CRC-SCs Through the MAPK Signaling Pathway

**Figure 4:**
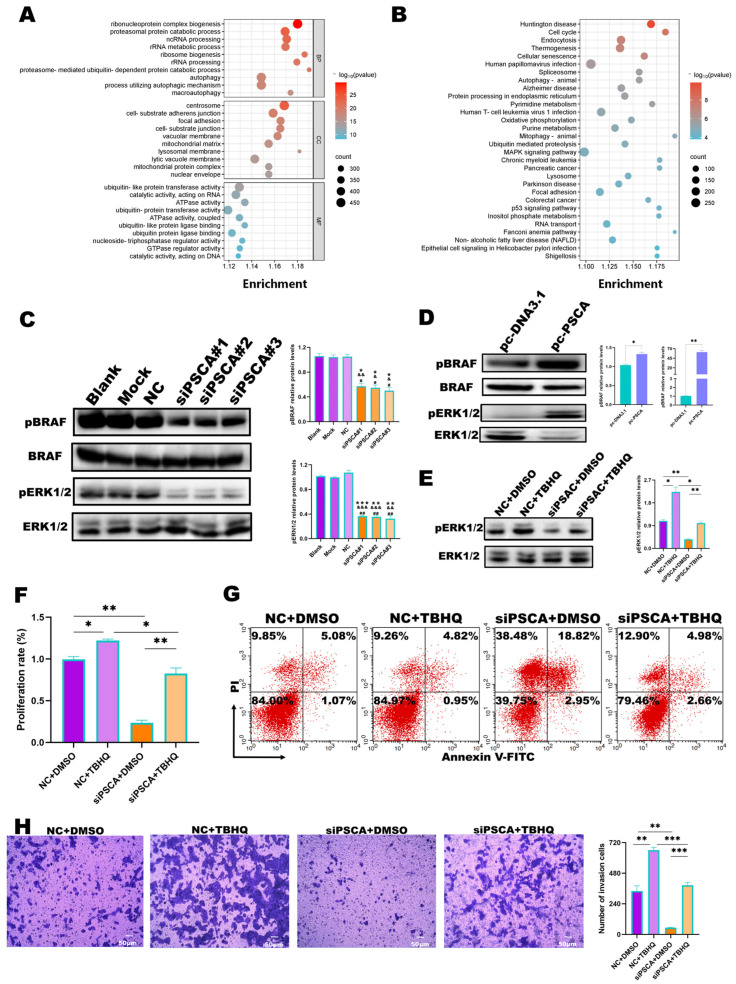
Mechanism exploration and functional verification of PSCA-directed modulation in CRC-SCs. (**A**) GO enrichment profiling of DEGs in HCT116SCs subjected to siPSCA or NC siRNA transfection. (**B**) KEGG enrichment of DEGs in HCT116SCs following siPSCA or NC siRNA transfection. (**C**) Phosphorylation levels of BRAF and ERK1/2 in HCT116SCs upon PSCA silencing (* *p* < 0.05, ** *p* < 0.01, *** *p* < 0.001 vs. Blank; & *p* < 0.05, && *p* < 0.01, &&& *p* < 0.001 vs. Mock; # *p* < 0.05, ## *p* < 0.01 vs. NC). (**D**) Phosphorylation levels of BRAF and ERK1/2 in SW620SCs upon PSCA ectopic expression (* *p* < 0.05, ** *p* < 0.01 vs. pcDNA3.1). (**E**) p-ERK1/2 abundance in HCT116SCs following siPSCA transfection in the presence or absence of tert-butylhydroquinone (TBHQ) (* *p* < 0.05, ** *p* < 0.01). (**F**) Proliferative capacity of HCT116SCs following siPSCA transfection with or without TBHQ supplementation (* *p* < 0.05, ** *p* < 0.01). (**G**) Apoptotic ratio of HCT116SCs following siPSCA transfection with or without TBHQ exposure. (**H**) Invasive capability of HCT116SCs following siPSCA transfection with or without TBHQ administration, as evaluated by Transwell assay (** *p* < 0.01, *** *p* < 0.001).

To explore the mechanism by which PSCA downregulation affects CRC-SCs, RNA sequencing was performed on HCT116SCs transfected with siPSCA or NC siRNA. Differentially expressed genes (DEGs) were identified and subjected to GO and KEGG enrichment analyses. GO analysis revealed significant enrichment of these DEGs in cellular components associated with focal adhesions, cell-substrate junctions, and adherens junctions (Figure 4A). Additionally, KEGG analysis showed that these DEGs were primarily enriched in the MAPK signaling pathway (Figure 4B). Western blotting further revealed that the phosphorylation levels of BRAF and ERK1/2 (key components in the MAPK pathway) were decreased after PSCA knockdown (Figure 4C). Conversely, PSCA overexpression in SW620SCs significantly increased p-BRAF and p-ERK1/2 levels (Figure 4D). These findings indicated that PSCA acts upstream of the MAPK pathway.

To further confirm the regulatory effect of PSCA on the MAPK signaling pathway, rescue experiments were performed using tert-butylhydroquinone (TBHQ), an ERK1/2 activator. Western blotting results exhibited that TBHQ treatment partially reversed the decrease in p-ERK1/2 induced by PSCA knockdown (Figure 4E) and partially restored the reduced proliferation and invasion capacities (Figure 4F,H). Similarly, TBHQ attenuated the increased apoptotic rate caused by PSCA knockdown (Figure 4G). Collectively, these results demonstrate that PSCA regulates the proliferation, apoptosis, and invasion of CRC-SCs via the MAPK/ERK signaling pathway.

Furthermore, DUSP4 was predicted to interact with both PSCA and ERK1/2 in the GeneMANIA database (http://genemania.org) (Supplementary Figure S1A). KEGG database analysis (https://www.kegg.jp/, accessed on 9 July 2026) further indicated that DUSP4 dephosphorylates and thereby inactivates ERK1/2 (Supplementary Figure S1B), suggesting that DUSP4-mediated ERK1/2 inactivation may underlie PSCA’s regulatory effect on the signaling pathway.

Further investigation revealed that PSCA regulates DUSP4 expression: Western blotting results demonstrated PSCA knockdown significantly upregulated DUSP4 expression (Supplementary Figure S2A), whereas PSCA overexpression downregulated DUSP4 (Supplementary Figure S2B). These results suggest that PSCA may regulates ERK1/2 phosphorylation through DUSP4.

### 3.5. siPSCA-Mediated Suppression of CRC Xenograft Tumor Progression

**Figure 5:**
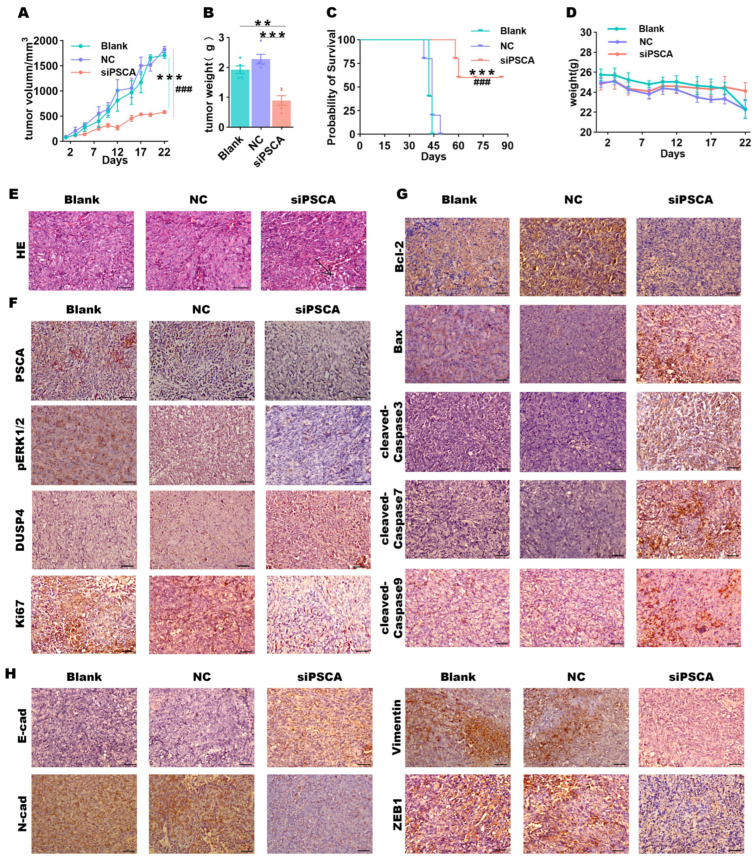
PSCA silencing suppresses xenograft tumorigenesis and prolongs overall survival of tumor-bearing mice. (**A**) In vivo tumor growth curves of HCT116SC xenografts in mice treated with 3-week siPSCA treatment (*** *p* < 0.001 vs. Blank; ### *p* < 0.001 vs. NC). (**B**) Terminal xenograft weights following 3-week siPSCA treatment (** *p* < 0.01, *** *p* < 0.001). (**C**) siPSCA treatment significantly prolongs the overall survival of HCT116SC-derived xenograft mice (*** *p* < 0.001 vs. Blank; ### *p* < 0.001 vs. NC). (**D**) Effects of 3-week siPSCA treatment on host body weight in tumor-bearing mice. (**E**) Hematoxylin and eosin (H&E) histomorphology of neoplasms. (**F**–**H**) Immunohistochemical profiling of xenograft tissue: (**F**) PSCA, pERK1/2, DUSP4, and Ki67; (**G**) apoptosis regulators (Bcl-2, Bax, cleaved caspase-3, cleaved caspase-7, and cleaved caspase-9); and (**H**) epithelial-mesenchymal transition (EMT) markers (E-cadherin, N-cadherin, vimentin, and ZEB1). Scale bar, 10 μm.

In vivo xenograft tumor models were established using HCT116-derived colorectal cancer stem cells (CR-CSCs) to clarify the functional role of PSCA knockdown in regulating CRC tumor progression. Consistent with the in vitro phenotypic changes, the in vivo tumor growth curve showed that the tumor growth rate in the siPSCA intervention group was markedly slower than that in the Blank and NC control groups during the entire observation period. After continuous treatment, the final tumor volume and tumor weight of xenografts in the siPSCA group were significantly decreased compared with those in the two control groups (Figure 5A,B), indicating that PSCA silencing effectively inhibits CR-CSC-derived tumor proliferation in vivo.

Survival analysis of tumor-bearing mice further demonstrated the protective effect of PSCA knockdown. All mice in the Blank and NC control groups died within the 60-day observation period. The siPSCA-treated mice exhibited a significantly prolonged survival time compared with the control mice (Figure 5C). Additionally, no significant differences in body weight were observed among the Blank, NC, and siPSCA groups throughout the animal experiment (Figure 5D), confirming that intratumoral siPSCA intervention possessed good biosafety and did not cause obvious systemic toxicity in nude mice.

Histopathological morphological changes of xenograft tumors were further observed via HE staining. Tumor tissues from the Blank and NC groups exhibited typical malignant pathological characteristics, including large and hyperchromatic nuclei, minimal cytoplasmic volume, dense and orderly tumor cell arrangement, and rare necrotic areas. In comparison, tumor tissues in the siPSCA group displayed obvious malignant phenotypic regression: tumor cells were loosely arranged with disordered tissue structure, accompanied by widespread nuclear pyknosis, nuclear fragmentation, and cytoplasmic vacuolation. In addition, increased inflammatory cell infiltration and large-scale necrotic lesions were extensively observed in siPSCA-treated tumor tissues (Figure 5E), suggesting that PSCA knockdown triggers tumor cell necrosis and suppresses the malignant proliferation of xenograft tumors.

IHC staining was performed to further verify the molecular regulatory mechanism of siPSCA in vivo. The results revealed that the protein expression level of PSCA was prominently downregulated in the siPSCA group, confirming the stable knockdown efficiency of PSCA in xenograft tumor tissues. Mechanistically, the phosphorylation level of ERK1/2 (pERK1/2), the core effector of the MAPK signaling pathway, was significantly decreased after PSCA silencing, whereas the expression of pERK1/2 dephosphorylation regulator DUSP4 was markedly upregulated (Figure 5F). Correspondingly, the expression of the proliferation biomarker Ki-67 was significantly reduced in the siPSCA group, indicating that PSCA knockdown efficiently represses in vivo tumor proliferative activity.

Furthermore, the apoptosis-related protein expression pattern was distinctly altered following PSCA inhibition. The expression of the anti-apoptotic protein Bcl-2 was significantly downregulated in siPSCA tumor tissues. In contrast, the levels of pro-apoptotic proteins, including Bax, cleaved-caspase-3, cleaved-caspase-7, and cleaved-caspase-9, were significantly elevated in the siPSCA group (Figure 5G), demonstrating that PSCA silencing facilitates tumor cell apoptosis in vivo.

Moreover, the EMT phenotype of xenograft tumors was notably suppressed after PSCA knockdown. IHC results showed that the expression of the epithelial marker E-cadherin was significantly upregulated in the siPSCA group. Meanwhile, the mesenchymal markers N-cadherin and vimentin and the EMT transcription factor ZEB1 were significantly downregulated (Figure 5H), indicating that siPSCA effectively blocks EMT progression and further restrains the invasive and metastatic potential of CRC xenograft tumors.

In summary, consistent with the in vitro findings, in vivo xenograft experiments confirmed that PSCA knockdown suppresses CR-CSC tumor growth, induces tumor necrosis and cell apoptosis, inhibits cell proliferation and EMT progression, and prolongs the survival of tumor-bearing mice via negative regulation of the MAPK signaling pathway. Collectively, these results demonstrate that PSCA acts as a critical oncogenic regulator during CRC progression.

## 4. Discussion

Genes belonging to the Ly6 family are localized on chromosome 8q24.3, a genomic region that frequently undergoes amplification of human oncogenes [26]. Previous research has demonstrated that specific members of the Ly6 family contribute to the progression of tumors [26,27,45]. Ly6a functions as a tumor-initiating biomarker in lung cancer [28] and plays a role in regulating the development of breast tumors as well as the migration of breast cancer cells [49,50]. Ly6d acts as a prognostic indicator for advanced prostate cancer, with its expression closely correlated with prostate cancer stem cells (CSCs) [51]. As an independent prognostic factor for colorectal cancer (CRC), Ly6e is closely related to the migration and invasion capabilities of CRC cells [32,33]. Additionally, Ly6k also facilitates the progression of ovarian and breast cancers [52,53].

As a member of the Ly6 family, PSCA has been previously recognized as a prognostic biomarker for bladder and gastric cancers [54,55]. In the present study, high expression of PSCA was observed in CRC-SCs, which prompted us to examine the impacts of siPSCA on the proliferation, invasion, and apoptosis of CRC-SCs. The results indicated that siPSCA treatment is capable of reducing CRC-SCs’ proliferation and invasion while enhancing CRC-SCs’ apoptosis. To clarify the molecular mechanism through which PSCA exerts its effects, RNA sequencing (RNA-Seq) was conducted. The analysis revealed that the differentially expressed genes in the groups treated with siPSCA were significantly enriched in the MAPK pathway, which is consistent with earlier findings showing that Lynx1 knockdown results in reduced pMEK expression within the MAPK pathway [56]. Further investigations confirmed that downregulating PSCA in HCT116SCs effectively lowered the expression levels of pBRAF and pERK1/2, while upregulating PSCA in SW620SCs notably elevated the expressions of these two proteins. As key components of the MAPK pathway, pBRAF and pERK1/2 have been well documented to promote the proliferation and migration of various types of cancer cells [57,58,59]. The in vivo xenograft immunohistochemistry (IHC) results also demonstrated that the expression of pERK1/2 in tumors from the siPSCA group was lower than that in the Blank and NC groups. Collectively, the current findings suggest that PSCA enhances CRC-SCs’ proliferation and invasion while inhibiting CRC-SCs’ apoptosis via the MAPK pathway. Furthermore, IHC results showed an elevated expression of the epithelial–mesenchymal transition (EMT) marker E-cad in tumors from mice treated with siPSCA, whereas the expressions of N-cad, vimentin, and ZEB1 were reduced. Similar observations have been reported, where downregulation of Ly6e inhibits EMT in non-small-cell lung cancer cells [60]. To verify the regulatory effect of PSCA on ERK1/2, TBHQ—an ERK1/2 activator that can upregulate pERK1/2 expression to promote cell proliferation, migration, and invasion—was used in related experiments [61,62]. Our results showed that TBHQ partially counteracted the effects of siPSCA, leading to increased proliferation and invasion as well as decreased apoptosis in HCT116SCs.

It is worth noting that PSCA knockdown significantly elevated both the apoptotic and necrotic levels of HCT116SCs. However, overexpression of PSCA did not cause any significant changes in the apoptotic and necrotic levels of SW620SCs. To date, there are no published reports that link Ly6 family members to tumor necrosis. Based on our experimental findings, the promotion of tumor necrosis by siPSCA may involve other signaling pathways, which requires further exploration. Focusing on apoptosis and necrosis, future studies ought to investigate the effects of ERK1/2 inhibitors on PSCA overexpression in SW620SCs. Moreover, a detailed investigation into the specific mechanism by which PSCA influences cell apoptosis in CRC-SCs through the MAPK pathway should be conducted in subsequent research. Overall, the in vivo and in vitro results of this study indicate that PSCA could serve as a potential therapeutic target for CRC, and siPSCA treatment may enhance the prognosis of CRC via the MAPK pathway.

Building on the above mechanistic findings, we discuss the translational value of PSCA-targeted therapy. Systematic research confirms PSCA is highly upregulated in multiple malignancies as a promising therapeutic target [45]. Preclinical PSCA-targeted ADCs and CAR immune cells exert specific anti-tumor effects on PSCA-high tumor cells with mild side effects [63,64]. Matching our observation of elevated PSCA in CRC-SCs, antibody-conjugated nanoparticles deliver siRNA to eliminate drug-resistant stem cells without systemic toxicity [65].

Our data indicate PSCA suppression exerts therapeutic effects on PSCA-overexpressing CRC. PSCA silencing inhibits MAPK/ERK1/2 activation to block CRC-SC proliferation, invasion, and EMT, and induces stem cell apoptosis to synergize with chemotherapy. Existing studies show sustained MAPK stabilizes c-Myc, while ERK1/2 upregulates Oct-4, Sox-2, and Nanog to sustain stemness and chemoresistance, which drive CRC recurrence [66,67]. Thus PSCA-mediated MAPK inhibition may downregulate these stemness regulators and improve chemosensitivity. Combining PSCA-targeted agents with MAPK or immune checkpoint inhibitors yields synergistic anti-tumor effects. Our findings provide a preclinical basis for therapies against PSCA-high CRC-SCs, though further translational research is needed for clinical transformation [45,68].

## 5. Conclusions

This paper systematically extends the present knowledge of the functional pathway mediated by PSCA, naturally leading to the conclusion that the results will provide a more solid theoretical basis for the diagnosis and treatment of colorectal cancer (CRC). It has clearly shown that an ERK1/2 activator promotes pERK1/2 expression in CRC stem cells (CRC-SCs), which in turn enhances cell proliferation and invasion, but it also appropriately points out that the next logical step is to investigate how ERK1/2 inhibitors regulate the MAPK pathway in CRC, as this will yield deeper insights into the role of PSCA in the MAPK pathway of CRC. Finally, a more complete framework for PSCA’s role in tumor development can be built from there.

**Figure 6:**
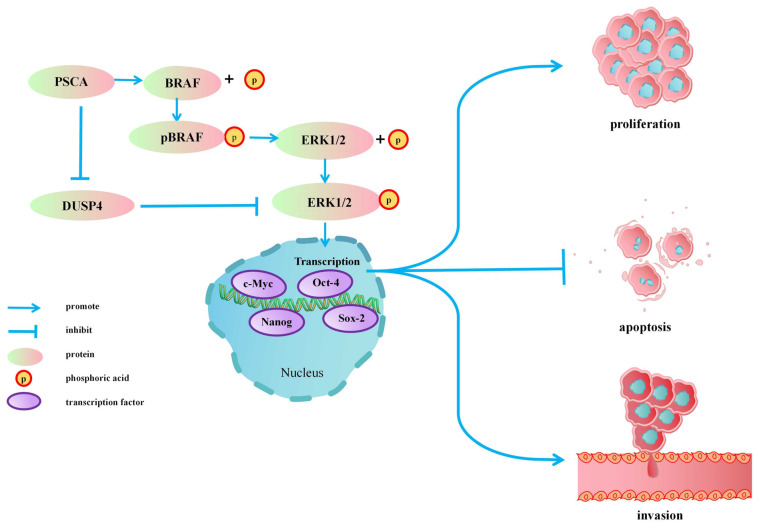
A schematic illustration of how PSCA influences CRC-SCs’ proliferation, apoptosis, and invasion at the molecular level.

The present paper clearly and convincingly shows that PSCA regulates the stemness of CRC-SCs with respect to proliferation, apoptosis, and invasion via the BRAF-ERK1/2 axis of the MAPK pathway (Figure 6). Therefore, it is reasonable to conclude that PSCA is a very promising therapeutic target for CRC.

## Data Availability

Raw RNA-seq data produced in this research have been uploaded to the NCBI GEO database with accession ID GSE337374. Other datasets generated and analyzed in this paper can be obtained from the corresponding author with reasonable application.

## Supplementary Materials

The following supporting information can be downloaded at: https://www.mdpi.com/article/10.3390/cimb48070737/s1.
