# Supplementary material for: The Oncogenic Role of Prostate Stem Cell Antigen (PSCA) in Colorectal Cancer: Implications for Targeted Therapy
Source: Curr Issues Mol Biol. 2026 Jul 20;48(7):737. doi: 10.3390/cimb48070737 (PMC13408367; doi:10.3390/cimb48070737)
Supplement: Supplementary file 1 [file cimb-48-00737-s001.zip › cimb-4381982-supplementary.pdf]

Supplementary Fig 1

A

B

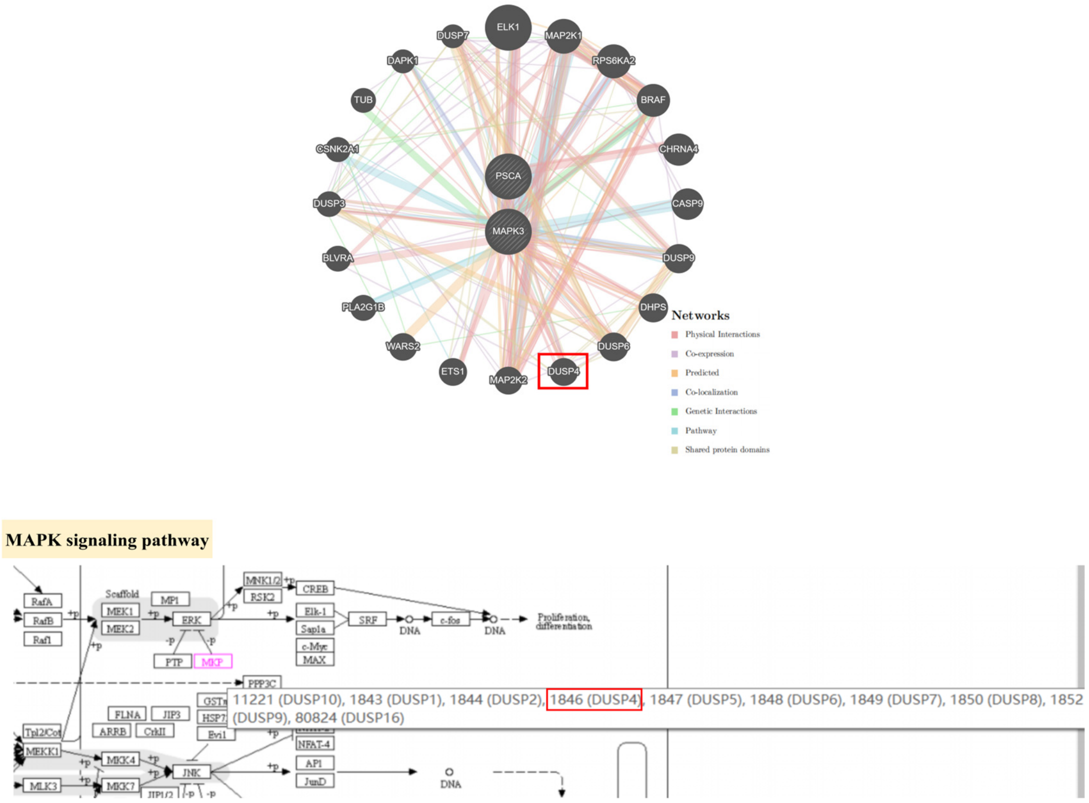

Supplementary Fig 1. Bioinformatics Investigation into the Associations Between PSCA and ERK1/2. (A) Analysis via the Genemania database demonstrated that DUSP4 interacts with both PSCA and ERK1/2 (formally designated as MAPK2). (B) KEGG pathway enrichment analysis illustrated that DUSP4 suppresses the phosphorylation status of ERK1/2.

Supplementary Fig 2

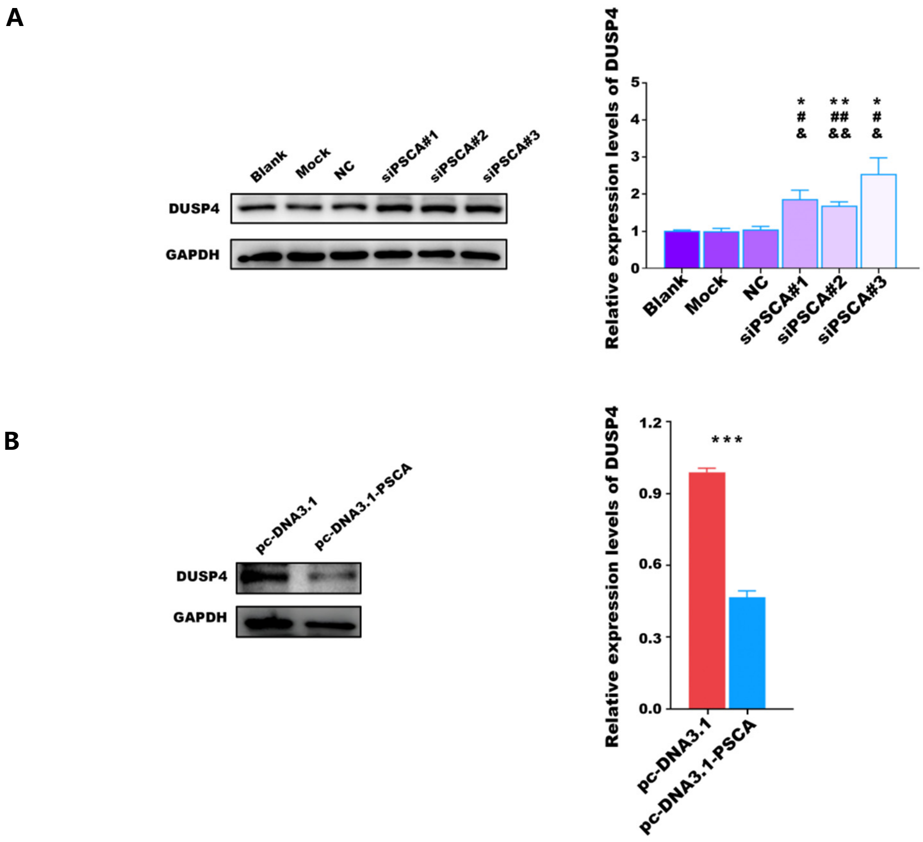

Supplementary Fig 2. PSCA regulates the expression of DUSP4. (A) Western blotting results of DUSP4 relative protein levels in HCT116SCs after down regulated PSCA (siPSCA) (*$p < 0.05$, **$p < 0.01$, compared with Blank; &$p < 0.05$, &&$p < 0.01$, compared with Mock; #$p < 0.05$, ##$p < 0.01$, compared with NC). (B) Western blotting results DUSP4 relative protein levels in SW620SCs after up regulated PSCA(***$p < 0.001$, compared with negative control).
